# Off-Target Effects of Drugs that Disrupt Human Mitochondrial DNA Maintenance

**DOI:** 10.3389/fmolb.2017.00074

**Published:** 2017-11-22

**Authors:** Matthew J. Young

**Affiliations:** Department of Biochemistry and Molecular Biology, Southern Illinois University School of Medicine, Carbondale, IL, United States

**Keywords:** nucleoside reverse transcriptase inhibitors, mitochondrial DNA polymerase gamma, human immunodeficiency virus (HIV), mitochondrial diseases, cancer, antiviral ribonucleosides, mitochondrial DNA (mtDNA)

## Abstract

Nucleoside reverse transcriptase inhibitors (NRTIs) were the first drugs used to treat human immunodeficiency virus (HIV) the cause of acquired immunodeficiency syndrome. Development of severe mitochondrial toxicity has been well documented in patients infected with HIV and administered NRTIs. *In vitro* biochemical experiments have demonstrated that the replicative mitochondrial DNA (mtDNA) polymerase gamma, Polg, is a sensitive target for inhibition by metabolically active forms of NRTIs, nucleotide reverse transcriptase inhibitors (NtRTIs). Once incorporated into newly synthesized daughter strands NtRTIs block further DNA polymerization reactions. Human cell culture and animal studies have demonstrated that cell lines and mice exposed to NRTIs display mtDNA depletion. Further complicating NRTI off-target effects on mtDNA maintenance, two additional DNA polymerases, Pol beta and PrimPol, were recently reported to localize to mitochondria as well as the nucleus. Similar to Polg, *in vitro* work has demonstrated both Pol beta and PrimPol incorporate NtRTIs into nascent DNA. Cell culture and biochemical experiments have also demonstrated that antiviral ribonucleoside drugs developed to treat hepatitis C infection act as off-target substrates for POLRMT, the mitochondrial RNA polymerase and primase. Accompanying the above-mentioned topics, this review examines: (1) mtDNA maintenance in human health and disease, (2) reports of DNA polymerases theta and zeta (Rev3) localizing to mitochondria, and (3) additional drugs with off-target effects on mitochondrial function. Lastly, mtDNA damage may induce cell death; therefore, the possibility of utilizing compounds that disrupt mtDNA maintenance to kill cancer cells is discussed.

## The origin of mitochondria and off-target effects of antibiotics

Mitochondria are best known for their role in generating energy by oxidative phosphorylation (OXPHOS), the process of coupling substrate oxidation to the production of the energy-rich molecule adenosine triphosphate (ATP). In addition to generating the bulk of the cell's energy supply mitochondria are important sites of calcium homeostasis, nucleotide and amino acid metabolism and biosynthesis of heme, iron-sulfur clusters, and ubiquinone. Mitochondria are eukaryotic organelles that share bacterial features such as a double-membrane structure and a circular multi-copied genome or mitochondrial DNA (mtDNA). The endosymbiotic theory hypothesizes mitochondria descended from an ancient alpha (α)-proteobacteria that developed a symbiotic relationship with an ancient nucleated cell (Gray, 2017). Support for the endosymbiotic hypothesis comes from striking similarities revealed between the mitochondrial and the *Rickettsia prowazekii* genomes (Andersson et al., 1998). Over time mitochondria lost most of their proto-bacterial genome to the nucleus. One thousand one hundred and forty-five nuclear-encoded mitochondrial gene products must be imported into mitochondria following translation on cytoplasmic ribosomes and estimates place the total mitochondrial proteome at ~1,500 gene products (Lopez et al., 2000; Calvo et al., 2016). Currently, there are ~170 known mitochondrial disease genes associated with ~500 clinical phenotypes suggesting that most medical specialists could see patients with mitochondrial disease (Scharfe et al., 2009; Turnbull and Rustin, 2016). The α-proteobacterial endosymbiont origin of mitochondria is supported by observations that certain antibiotics have off-target effects on mitochondrial ribosomes. Similar to bacterial translation, mitochondrial translation is initiated with an *N-*formylmethionine and mitochondrial but not cytoplasmic translation is sensitive to bacterial antibiotics such as chloramphenicol (CAP) and aminoglycosides (Wallace et al., 1975; Oliver and Wallace, 1982; Wallace and Chalkia, 2013). Additionally, mitochondrial ribosomes are resistant to inhibitors of eukaryotic translation such as emetine and cycloheximide (Oliver and Wallace, 1982).

## Mitochondrial disorders and the importance of mtDNA maintenance in human health

The haploid human nuclear genome consists of ~3 billion base pairs (bp) of DNA and contains ~20,000 protein-coding genes and ~23,000 non-coding genes. Examples of non-coding genes include transfer RNA (tRNA), ribosomal RNA (rRNA), micro RNA (miRNA), miscellaneous RNA (miscRNA), small nucleolar RNA (snoRNA), small nuclear RNA (snRNA), small cytoplasmic RNA (scRNA), and long non-coding RNA (lncRNA). In comparison, the mitochondrial genome harbors only 13 genes for polypeptides, 2 genes for rRNA, and 22 genes for tRNA on ~16,600 bp and mutations associated with maternally-inherited mitochondrial disorders have been identified in all 37 open reading frames. Similar to practically all prokaryotic genes, human mtDNA genes lack introns. *The 13 polypeptide-encoded genes code for subunits of the mitochondrial inner membrane (MIM) OXPHOS machinery*. While the size and coding capacity of mtDNA is much less than the nuclear genome our maternally inherited genome is critical to cellular viability as exemplified by the numerous disease mutations associated with it and by observations that knocking out mtDNA maintenance genes results in embryonic lethality in various mouse models (Park and Larsson, 2011). Currently, greater than 660 mtDNA mutations are associated with disease phenotypes (www.mitomap.org). The most common encephalopathies caused by mtDNA point mutations include Leigh Syndrome, Leber Hereditary Optic Neuropathy, MERRF (myoclonic epilepsy with ragged red fibers), MIDD (maternally inherited diabetes and deafness), MELAS (mitochondrial encephalomyopathy, lactic acidosis, and stroke-like episodes), non-syndromic hearing loss, and NARP (neuropathy, ataxia, retinitis pigmentosa) (Pinto and Moraes, 2014). Maintenance of the mitochondrial genome is also required to avoid apoptosis induced by mtDNA damage (Santos et al., 2003; Tann et al., 2011).

Molecules of mtDNA associate with various DNA-binding proteins on the matrix-side of the MIM and form protein-mtDNA structures known as nucleoids (Bogenhagen et al., 2008; Brown et al., 2011; Hensen et al., 2014; Young et al., 2015). Utilizing live-cell fluorescence microscopy or immunocytochemistry, nucleoids can be visualized as foci or puncta. Furthermore, a single cell can contain several thousand copies of mtDNA which are distributed within hundreds of individual mitochondria or throughout an elaborate mitochondrial reticular network (Miller et al., 2003; Spelbrink, 2010; Archer, 2013; Young et al., 2015). Localization of mtDNA at the MIM is likely important to coordinate mtDNA replication and transcription with mitochondrial translation, cytoplasmic translation, and mitochondrial protein import and assembly (Iborra et al., 2004; Spelbrink, 2010). Nuclear-encoded mitochondrial transcription machinery is imported into the organelle to transcribe mtDNA genes. Nuclear-encoded mitochondrial ribosomal subunits assemble with mtDNA-encoded rRNAs following protein import to form the translation machinery necessary to synthesize the 13 mtDNA-encoded polypeptides. Therefore, the MIM OXPHOS energy-generating process is strictly dependent on mtDNA maintenance and pharmacological blocks to mitochondrial genome replication would be devastating to this energy-generating process.

## Mitochondrial reactive oxygen species (ROS) and base excision repair (BER)

**Figure 1:**
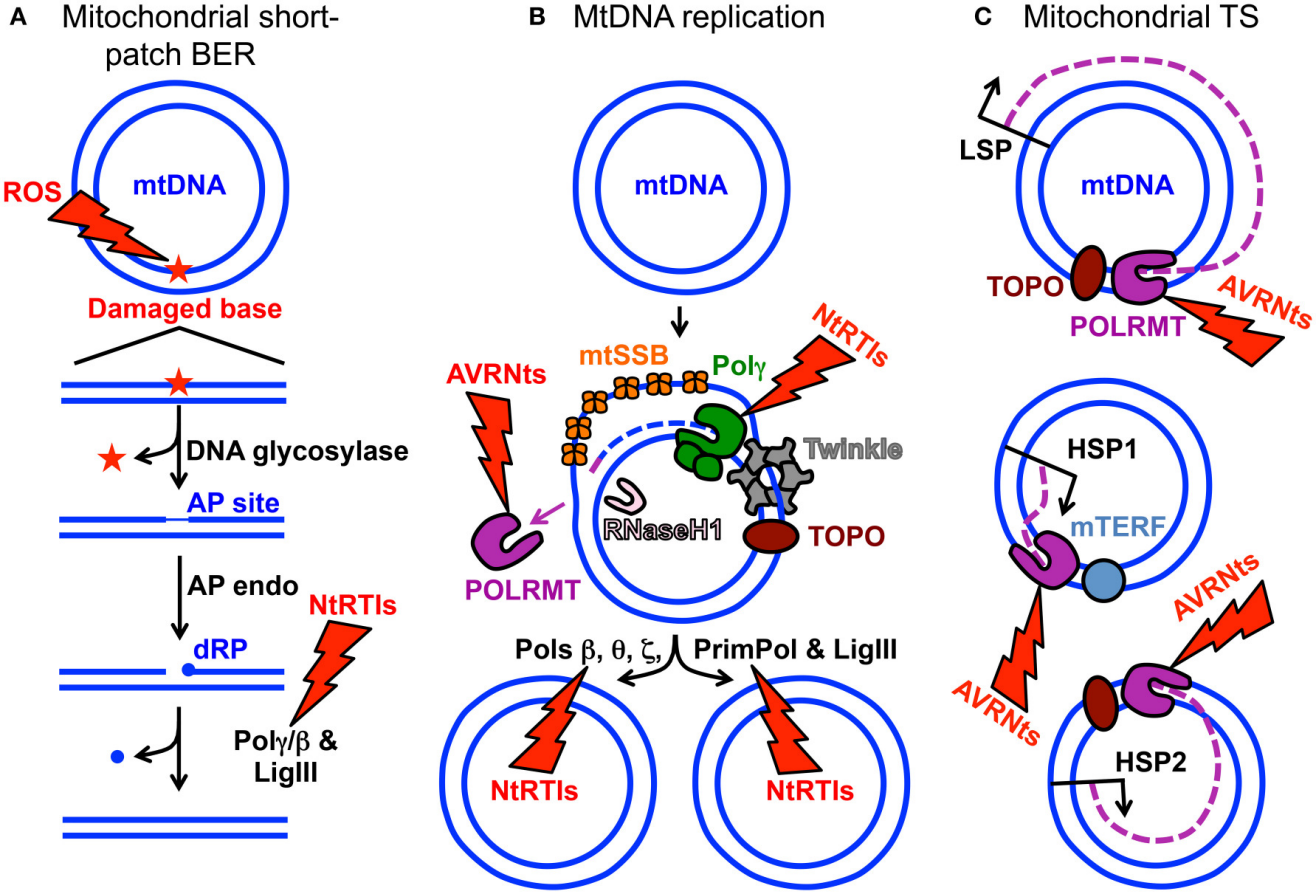
MtDNA maintenance and mitochondrial gene expression. **(A)** Mitochondrial short-patch base excision repair (BER) initiated with a monofunctional DNA glycosylase. The ROS lightning bolt represents reactive oxygen species-induced mtDNA damage generating an oxidized base lesion (star) that is subsequently removed and repaired by the BER machinery. Two blue circles represent the double-stranded circular mitochondrial genome. A region of the damaged mtDNA is shown below the circular genome to emphasize the BER pathway steps. AP site, apurinic/apyrimidinic site; AP endo, AP endonuclease; dRP, 5′-deoxyribose phosphate; Polγ/β the replicative mtDNA polymerase gamma or DNA polymerase beta; LigIII, mitochondrial DNA ligase III. The NtRTIs lightning bolt represents nucleotide reverse transcriptase inhibitors blocking Polγ or β. **(B)** Key components of the mtDNA replication and repair machinery. The small purple line represents an RNA primer while the blue dashed line represents newly synthesized mtDNA. TOPO, topoisomerase; Twinkle, Twinkle mtDNA helicase; POLRMT, mitochondrial RNA polymerase and primase; RNaseH1, Ribonuclease H1; mtSSB, mitochondrial single-stranded DNA binding protein. DNA polymerase beta, theta, zeta, and the DNA *prim*ase and translesion DNA *pol*ymerase are represented by Pols β, θ, ζ, and PrimPol respectively. These polymerases likely assist Polγ with overcoming mtDNA damage. The AVRNts lightning bolt represents antiviral ribonucleotides blocking POLRMT activity. **(C)** Polycistronic mitochondrial transcription. Mitochondrial transcription (TS) occurs from three promoters: (1) LSP, light-strand promoter, (2) HSP1, heavy-strand promoter 1, and (3) HSP2, heavy-strand promoter 2. Three purple dashed lines represent transcripts synthesized from the promoters. Although not visualized in the cartoon, mitochondrial TS initiation requires mitochondrial TS factor A (TFAM) and either of mitochondrial TS factors B1 or B2 (TFB1M or TFB2M). It is generally accepted that TFB2M is the primary factor for TS initiation (Shutt et al., 2010). Mitochondrial TS termination factor is represented by mTERF.

Aberrant electron leakage from the OXPHOS machinery to molecular oxygen (O_2_) can generate reactive oxygen species (ROS) which, if not detoxified, cause damage to intracellular molecules such as DNA, RNA, lipids, and proteins (Wallace, 2005). The close proximity of mtDNA-containing nucleoids to the OXPHOS machinery generating ROS has been suggested to inflict more damage on the mitochondrial genome than on the nuclear genome (Tann et al., 2011). ROS-induced DNA damage includes a large quantity of mutagenic oxidized bases and the mutation rate of human mtDNA has been estimated to be 20–100-fold higher relative to nuclear DNA. Nuclear-encoded base excision repair (BER) machinery is imported into the mitochondrion to assist with mending abnormal and oxidized base lesions. During mitochondrial short-patch BER, an oxidized base may first be excised by a monofunctional DNA glycosylase such as UNG1 or MUTYH, Figure 1A. DNA glycosylase cleaves the damaged base *N*-glycosidic bond generating an abasic or apurinic/apyrimidinic (AP) site then this site is cleaved by an AP endonuclease to generate a 3′-OH and non-ligatable 5′-deoxyribose phosphate (dRP) moiety. Next, the catalytic subunit of the replicative mitochondrial 5′-3′ DNA polymerase gamma (Polγ) fills in the gap via its DNA polymerase activity and removes the dRP group via its 5′-deoxyribose phosphate (dRP) lyase activity leaving a 5′-phosphate. Lastly, DNA ligase III seals the nick and the damage is repaired (Longley et al., 1998). Alternatively, a bifunctional DNA glycosylase harboring an intrinsic lyase activity can cleave the *N*-glycosidic bond and incise the AP site; however, the ends generated by the incision are non-ligatable and must be processed by either AP endonuclease or polynucleotide kinase 3′-phosphatase then Polγ can fill the gap and ligase can seal the nick (Bebenek and Kunkel, 2004; Alexeyev et al., 2013). Figure 1A is a simplified cartoon of short-patch BER. Details regarding mitochondrial short-patch and long-patch BER pathways have been thoroughly reviewed (Alexeyev et al., 2013; Copeland and Longley, 2014; Van Houten et al., 2016).

## Polγ and the replisome

Human Polγ is the replicative mitochondrial DNA polymerase that harbors 3′-5′ exonucleolytic proofreading activity and participates in mtDNA repair (Young and Copeland, 2016). Polγ is a heterotrimer consisting of one 140-kDa catalytic subunit, p140 encoded by the nuclear *POLG* gene, and a 110-kDa homodimeric processivity subunit, p55 encoded by the nuclear *POLG2* gene. MtDNA disorders can be caused by genetic defects in nuclear genes, and a class of genes specifically linked to instability of mtDNA has emerged over the last 16 years which includes *POLG* and *POLG2*, Table 1 (Young and Copeland, 2016). Nuclear mitochondrial disease genes are associated with a complex spectrum of early onset and late onset type phenotypes. One subclass of disorders, mtDNA depletion syndromes, may arise due to defects in genes encoding mtDNA replication machinery (ex. *POLG*, Alpers-Huttenlocher syndrome) or enzymes required for nucleotide synthesis (ex. *TK2*). MtDNA depletion syndromes in of themselves are variable and clinical manifestations may include myopathy, encephalomyopathy, neurogastrointestinal, or hepatocerebral phenotypes (Stiles et al., 2016). In addition to the 5′-3′ DNA polymerase, 3′-5′ exonuclease, and 5′ dRP lyase activities mentioned above the p140 catalytic subunit harbors reverse transcriptase (RT) activity (Murakami et al., 2003; Kaguni, 2004; Graziewicz et al., 2006). The RT activity or RNA-dependent DNA polymerase activity is similar to viral enzymes such as human immunodeficiency virus RT (HIV-RT). Unfortunately, as will be discussed below, biochemical experiments have demonstrated that Polγ is sensitive to inhibition by metabolically active forms of anti-HIV nucleoside reverse transcriptase inhibitors (NRTIs) known as nucleotide reverse transcriptase inhibitors (NtRTIs). Treatment of HIV-infected patients with NRTIs is accompanied by loss of mitochondrial function and NRTI toxicity mimics mitochondrial genetic diseases and induces similar symptoms such as mtDNA depletion (Graziewicz et al., 2006). One explanation as to why Polγ harbors RT activity may be to replicate past ribonucleotides (ribonucleoside monophosphates) that are evenly distributed between the two strands of mtDNA (Murakami et al., 2003; Berglund et al., 2017). The homodimeric Polγ p55 subunit imparts high processivity onto the holoenzyme by increasing the binding affinity to DNA (Lim et al., 1999; Young et al., 2015). Processivity is a measurement of the extent of Polγ DNA synthesis during a primer-template binding event. Polγ functions in conjunction with several replisome components including: (1) topoisomerase, (2) mitochondrial single-stranded DNA binding protein (mtSSB), (3) Twinkle mtDNA helicase, (4) RNaseH1, (5) mitochondrial RNA polymerase (POLRMT), and (6) mitochondrial DNA ligase III, Figure 1B. Additional factors critical for mitochondrial genome maintenance include: the multifunctional mitochondrial transcription factor A (TFAM) with significant roles in mtDNA replication and packaging, the RecB-type mitochondrial genome maintenance 5′-3′ exonuclease 1 (MGME1), the RNA and DNA 5′ flap endonuclease (FEN1), and the helicase/nuclease, DNA2 (Kalifa et al., 2009; Kornblum et al., 2013; Ngo et al., 2014). MGME1, FEN1, and DNA2 have all been implicated in mtDNA BER (Copeland and Longley, 2014). Furthermore, DNA2 has been shown to stimulate Polγ activity and to co-localize with Twinkle in the mitochondrial nucleoid, which suggests an important role in the replisome (Zheng et al., 2008). Some of the genes encoding components of the mtDNA replication machinery may have been acquired as part of a protomitochondrial genome, in the form of integrated phage genes from a T-odd lineage, which were then transferred to the eukaryotic nucleus (Shutt and Gray, 2006). This hypothesis is based on the shared conservation of primary protein amino acid sequences of T-odd bacteriophages with mitochondrial Polγ, POLRMT, and Twinkle helicase.

**Table 1:** Nuclear genes identified in mitochondrial patients that affect mtDNA stability^a^.

**Gene**	**Disorder^b^**	**Chromosomal locus**	**Function**
**MtDNA REPLICATION AND REPAIR**			
*POLG*	PEO/Alpers/ataxia	15q25	Polγ catalytic subunit
*POLG2*	PEO	17q	Polγ processivity subunit
*Twinkle*	PEO/ataxia	10q24	MtDNA helicase
*RNASEH1*	PEO/ataxia	2p25	Mitochondrial and nuclear RNaseH1 (Reyes et al., 2015)
*DNA2*	PEO	10q21.3-22.1	Mitochondrial and nuclear helicase/nuclease (Ronchi et al., 2013)
*MGME1*	PEO, mtDNA depletion	20p11.23	RecB type exonuclease
*TFAM*	Neonatal liver failure mtDNA depletion	10q21.1	Mitochondrial transcription factor A (Stiles et al., 2016)
**MAINTAINING dNTP POOLS**			
*ANT1*	PEO	4q35	Adenine nucleotide translocator
*TP*	MNGIE	22q13.33	Thymidine phosphorylase
*DGUOK*	MtDNA depletion	2p13	Deoxyguanosine kinase
*TK2*	MtDNA depletion	16q22-23.1	Mitochondrial thymidine kinase
*SUCLA2*	MtDNA depletion	13q14.2	ATP-dependent Succinate-CoA ligase
*SUCLG1*	MtDNA depletion	2p11.2	GTP-dependent Succinate CoA ligase
*RRM2B*	MtDNA depletion	8q23.1	p53-Ribonucleotide reductase, small subunit
*MPV17*	MtDNA depletion and deletion	2p23.3	Mitochondrial inner membrane protein
*ABAT*	MtDNA depletion	16p13.2	4-aminobutyrate aminotransferase (Besse et al., 2015)
**MITOCHONDRIAL HOMEOSTASIS AND DYNAMICS**			
*OPA1*	Dominant optic atrophy	3q29	Dynamin-related GTPase
*MFN2*	Recessive optic atrophy	1p36.22	Mitofusin 2 (Rouzier et al., 2012)
*FBXL4*	MtDNA depletion, Encephalopathy	6q16.1-16.3	Mitochondrial LLR F-Box protein

a *The table is an updated version of Table 1 found in reference (Young and Copeland, 2016) and is reproduced with permission*.

b *PEO, progressive external ophthalmoplegia; MNGIE, mitochondrial neurogastrointestinal encephalomyopathy*.

In agreement with the requirement for mtDNA replication re-initiation between embryonic day (E)6 and 7.5 (Stewart and Larsson, 2014), p140 in animal cells was shown to be essential using *POLG* knockout (KO) mice. The *POLG* KO results revealed embryonic lethality at E7.5–8.5 with subsequent depletion of mtDNA (Hance et al., 2005). Comparatively, several studies have illustrated the essential role of p55 in mtDNA replication: (i) two separate null mutations in the *Drosophila melanogaster POLG2* gene lead to lethality in the early pupal stage of fly development (Iyengar et al., 2002), (ii) homozygous *POLG2* KO mice are embryonic lethal at E8–8.5 (Humble et al., 2013), and (iii) in a porcine oocyte knockdown model, oocyte maturation requires *POLG2* (Lee et al., 2015). Mouse RNaseH1^−/−^ embryos are null at E8.5 and have decreased mtDNA content leading to apoptotic cell death (Cerritelli et al., 2003). A mouse model of Twinkle deficiency has been generated by transgenic expression of a Twinkle cDNA with an autosomal dominant mutation found in patients (Tyynismaa et al., 2005; Tyynismaa and Suomalainen, 2009). These mice developed progressive respiratory chain deficiency at 1 year of age in cerebellar Purkinje cells, hippocampal neurons, and skeletal muscle. The affected cells accumulated multiple mtDNA deletions. These “Deletor” mice recapitulate many of the symptoms associated with PEO and represent a useful research model.

## Newly identified human DNA polymerases localizing to mitochondria - pols β, θ, ζ, and PrimPol

Prior to 2013 human Polγ was the only polymerase out of the 17 known cellular DNA polymerases demonstrated to localize to human cell mitochondria; however, mounting evidence suggests it is not the only one. Recently, DNA polymerase beta (Polβ) was detected in mitochondrial extracts prepared from human embryonic kidney cells (HEK-293T) and from various tissues obtained from mice (Sykora et al., 2017). Analysis of mouse tissue extracts revealed Polβ in brain and kidney mitochondria while none was detectable in heart, liver, or muscle. As a key member of the nuclear BER machinery Polβ provides the majority of the required 5′-dRP lyase activity in the nucleus; therefore, Polβ may participate in mitochondrial BER. In a short-patch BER scenario, following the actions of a monofunctional DNA glycosylase and an AP endonuclease Polβ could insert a nucleotide onto the 3′-OH then remove the 5′-dRP group using its dRP lyase activity followed by the nick sealing action of DNA ligase (Figure 1A). As mentioned above mitochondrial ribosomes are sensitive to CAP (CAP^S^). MtDNA can develop resistance to CAP (CAP^R^) through mutation of the mtDNA 16S rRNA gene changing the specificity of CAP for the mitochondrial ribosome and inhibiting its binding (Blanc et al., 1981; Kearsey and Craig, 1981; Wallace and Chalkia, 2013; Sykora et al., 2017). In two HEK-293T Polβ KO cell lines very few CAP^R^ cells could be isolated relative to the parental cell line when plated at high cell density. This finding suggests that Polβ may mediate mtDNA mutational events. Utilizing *in vitro* biochemistry Polβ was also demonstrated to interact with the mitochondrial Twinkle helicase and this interaction facilitated Polβ strand displacement. Enhanced strand displacement suggests Polβ may participate in the mitochondrial long-patch BER pathway (Sykora et al., 2017). As many *Twinkle* gene-disease mutations result in protein variants with partial helicase defects (Longley et al., 2010) it would be interesting to investigate strand displacement using recombinant Twinkle disease variants and Polβ to provide insight into possible mechanisms of *Twinkle-*related mitochondrial disease. Besides BER, other roles of Polβ in mtDNA maintenance remain to be elucidated. Polβ is not likely a replicative mtDNA polymerase as this enzyme lacks 3′-5′ exonuclease proofreading activity, has low processivity, incorporating few nucleotides each time it binds a primer-template, and has a high error rate relative to the proofreading proficient Polγ (Bebenek and Kunkel, 2004). However, *POLG*-related disease mutations that abolish p140 activity and are associated with late age of onset may argue in favor of redundant DNA polymerase function(s) in human cell mitochondria (Sykora et al., 2017). Polβ^−/−^ mouse embryos survive the course of development but die immediately at the perinatal stage suggesting the cause of death is a neonatal respiratory defect (Sugo et al., 2000).

The DNA *prim*ase and translesion DNA *pol*ymerase, PrimPol, has been identified in mitochondria isolated from HEK-293T cells (García-Gómez et al., 2013). Translesion DNA polymerases are specialized enzymes that pass through DNA damage. However, PrimPol is likely only required for mtDNA repair and not for mtDNA replication, as *PRIMPOL*^−/−^ KO mice are viable. Like Polβ PrimPol is localized to both the nucleus and the mitochondrion and lacks proofreading activity. Of note to human genetic disease, mutation of *PRIMPOL* is associated with the ocular disorder high myopia (Zhao et al., 2013; Keen et al., 2014). DNA polymerase theta (Polθ) was recently identified in mitochondria isolated from human cells (Wisnovsky et al., 2016). Polθ is a proofreading-deficient and error-prone polymerase capable of translesion DNA polymerization (Arana et al., 2008). In the nucleus, Polθ is implicated in double-strand DNA break repair, non-homologous end joining and maintenance of DNA replication timing. The translesion DNA polymerase zeta (Polζ) is composed of two subunits the catalytic subunit Rev3 and the structural subunit Rev7. To date, no evidence for Rev7 localization to human cell mitochondria has been described but the Rev3 subunit has been reported to localize to the organelle and may play a role in protecting mtDNA from ultraviolet radiation-induced DNA damage (Singh et al., 2015). Compared to Polγ, Pols θ and ζ localize to both the nucleus and the mitochondrion, have low fidelity, lack proofreading activity and have only moderate processivity (Bebenek and Kunkel, 2004; Arana et al., 2008; Lee et al., 2014); therefore, their main roles are likely in assisting the core replisome in overcoming mtDNA damage. Polθ KO mice are viable whereas Polζ KO mice are embryonic lethal with a block in embryo development not beyond 8–8.5 days *post-coitus* (Esposito et al., 2000; Shima et al., 2004). Details regarding the evidence supporting mitochondrial localization of the aforementioned human DNA polymerases have been reviewed (Krasich and Copeland, 2017).

## Nucleoside reverse transcriptase inhibitors, NRTIs

NRTIs were the first drugs used to treat HIV, the cause of acquired immunodeficiency syndrome (AIDS). NRTIs remain effective today for treating HIV when combined with other drugs. Highly active antiretroviral therapy (HAART) uses multiple drugs to act on different HIV life-cycle stages. For patients with HIV infection, HAART regimens include treatment with NRTIs in combination with non-nucleoside reverse transcriptase inhibitors (NNRTIs) or protease inhibitors, PIs (Nolan and Mallal, 2004). NNRTIs and NRTIs primarily block HIV genome replication by inhibiting the HIV-RT from transcribing the viral single-stranded RNA genome into DNA. FDA-approved NRTIs used to treat HIV infection include: ddC (zalcitabine), 3TC (Epivir®, lamivudine), AZT (Retrovir®, zidovudine), ddI (Videx-EC®, didanosine), PMPA (Viread®, tenofovir DF), d4T (Zerit®, stavudine), ABC (Ziagen®, abacavir), and FTC (emtricitabine, Emtriva®), Table 2. NRTIs may be administered to patients in fixed-dose combinations: Combivir® (Retrovir + Epivir), Descovy® (tenofovir alafenamide + Emtriva), Epzicom® (Epivir + Ziagen), Trizivir® (Retrovir + Epivir + Ziagen), and Truvada® (Viread + Emtriva), https://www.hiv.va.gov/patient/treat/NRTIs.asp. The history of antiretroviral drugs and the currently used antiretroviral therapies have been reviewed (Pau and George, 2014). A discussion of what is currently known regarding NRTIs with off-target effects on mtDNA replication is discussed below.

**Table 2:** NRTIs with off-target effects on human DNA polymerases that localize to mitochondria.

**Drug**	**Target**	**Potential off-target^a^**	**Mode of action**	**Side effects/toxicity/other notes**	**Experimental evidence for off-target effect**	**References**
ddC, 2′, 3′-dideoxycytidine, zalcitabine, hivid	HIV-RT	Polγ, PrimPol, Polβ	Deoxycytidine analog, chain-terminator	Peripheral neuropathy, sensorineural deafness, hypertrophic cardiomyopathy; according to the FDA ddC is no longer marketed	MtDNA depletion in various human cell lines; efficiently incorporated by Polγ and PrimPol, *in vitro* (Polγ 14-fold reduction in dCTP/ddCppp discrimination relative to PrimPol); Polβ incorporates and sensitive to ddCppp inhibition *in vitro*	Martin et al., 1994; Pelletier et al., 1994; Johnson et al., 2001; Lewis et al., 2001; Lim and Copeland, 2001; Birkus et al., 2002; Ashley et al., 2005; Setzer et al., 2005; Rocher et al., 2008; Jemt et al., 2015; Mislak and Anderson, 2015
ddI, 2′, 3′-dideoxyinosine, didanosine, Videx-EC®	HIV-RT	Polγ, PrimPol, Polθ?	Deoxyadenosine analog, chain-terminator	Peripheral neuropathy, pancreatitis, hypertrophic cardiomyopathy, diabetes mellitus, hepatocellular failure, lactic acidosis; ddI is metabolized to ddAppp	Aberrant cristae and decreased mtDNA copy number in human cell lines; ddAppp (active form of ddI) incorporated efficiently by Polγ and incorporated by PrimPol *in vitro* (Polγ 233-fold reduction in dATP/ddAppp discrimination relative to PrimPol)	Medina et al., 1994; Johnson et al., 2001; Lewis et al., 2001; Birkus et al., 2002; Setzer et al., 2005; Mislak and Anderson, 2015; Zahn et al., 2015
d4T, 2′, 3′-didehydro-2′, 3′-dideoxythymidine, stavudine, Zerit®	HIV-RT	Polγ, Polβ	Thymidine analog, chain-terminator	Peripheral neuropathy, pancreatitis, hepatocellular failure, lactic acidosis, lipodystrophy; no longer recommended for administration	Aberrant cristae and decreased mtDNA copy number in human cell lines; incorporated efficiently by Polγ *in vitro*; Polβ incorporates and sensitive to d4Tppp inhibition *in vitro*	Martin et al., 1994; Vázquez-Acevedo et al., 1995; Johnson et al., 2001; Lewis et al., 2001; Lim and Copeland, 2001; Birkus et al., 2002; Setzer et al., 2005; Koczor and Lewis, 2010
3TC, 2′, 3′-dideoxy-3′-thiacytidine, lamivudine, Epivir®	HIV-RT	Polγ, Polβ	Zalcitabine/cytosine analog (see above), chain-terminator	Peripheral neuropathy, lactic acidosis, hepatomegaly with steatosis	Kinetic analysis with HeLa Polγ, modest inhibition of Polγ *in vitro*; Polβ has a 9-fold reduction in dCTP/L-3TCppp discrimination in comparison to Polγ *in vitro*	Johnson et al., 2001; Lewis et al., 2001; Lim and Copeland, 2001; Koczor and Lewis, 2010; Brown et al., 2011
PMPA, (*R*)-9- (2-phosphonylmethoxypropyl)adenine, TFV, tenofovir	HIV-RT	Polγ, Polβ	Deoxyadenosine monophosphate analog, chain-terminator	Mitochondrial nephrotoxicity, kidney dysfunction; Viread®/TDF is a prodrug of PMPA	Modest inhibition of Polγ *in vitro*; Polβ has a 270-fold reduction in dATP/PMPApp discrimination in comparison to Polγ *in vitro*	Johnson et al., 2001; Koczor and Lewis, 2010; Brown et al., 2011
AZT, 3′-azido-2′, 3′-dideoxythymidine, zidovudine, ZDV, Retrovir®	HIV-RT	Polγ, PrimPol, Polβ	Thymidine analog, chain-terminator, decreases levels of pyrimidines	Myopathy including ragged red fibers, decreased muscle mtDNA, bone marrow suppression, hypertrophic cardiomyopathy, sideroblastic anemia, pancytopenia, hepatocellular failure, lactic acidosis	Decreased mtDNA in cell culture, biochemical defects with Polγ *in vitro*; modestly incorporated by Polγ; Polβ has an ~3,850-fold reduction in dTTP/AZTppp discrimination relative to Polγ and PrimPol has an ~60-fold reduction in dTTP/AZTppp discrimination relative to Polγ *in vitro*.	Johnson et al., 2001; Lewis et al., 2001; Lim and Copeland, 2001; Brown et al., 2011; Mislak and Anderson, 2015; Fernández-Moreno et al., 2016
CBV, (−)-cis-2-amino-1,9-dihydro-9-(4-hydroxymethyl)-(2-cyclopenten-1-yl)-6H-purin-6-one, carbovir active form of abacavir, ABC, see below	HIV-RT	Polγ, PrimPol	Deoxyguanosine analog, chain-terminator	See below	Strongly incorporated by PrimPol *in vitro* and modest inhibition of Polγ *in vitro*	Johnson et al., 2001; Lewis et al., 2001; Lim and Copeland, 2001; Mislak and Anderson, 2015
ABC, [(1S,4R)-4-[2-amino-6-(cyclopropylamino)-9-purinyl]-1-cyclopent-2-enyl]methanol, abacavir, Ziagen®	HIV-RT	Polγ, PrimPol	Deoxyguanosine analog, chain-terminator	Increased myocardial infarction and congestive heart failure; Note: following intracellular phosphorylation ABC monophosphate is converted to CBV monophosphate by cytosolic enzymes then to CBVppp by cellular kinases	See CBV above	Koczor and Lewis, 2010; Mislak and Anderson, 2015
FIAU, 1-(2-deoxy-2-fluoro-β-D-arabinofuranosyl)-5-iodouracil, fialuridine, fluoroiodoarauridine	Hepatitis B, herpes virus DNA pols	Polγ	Uridine analog, not a chain terminator as it contains a 3′ OH, but impairs DNA elongation at adenosine tracts	Severe lactic acidosis, liver failure, and steatosis, kidney failure, myopathy, peripheral neuropathy; discontinued use due to severe hepatotoxicity and death	Inhibition of Polγ *in vitro*, cytotoxic to human Molt-4 cells, aberrant mitochondrial structures	Martin et al., 1994; McKenzie et al., 1995; Lewis et al., 1996, 2001; Johnson et al., 2001
FTC, 5-fluoro-1-[(*2R*,5*S*)-2-(hydroxymethyl)-1,3-oxathiolan-5-yl]cytosine, RCV, emtricitabine, Emtriva®, coviracil, racivir	HIV-RT	Polγ, Polβ	Deoxycytidine analog, chain termination	Lactic acidosis, hepatomegaly with steatosis	Polβ has a 100-fold reduction in dCTP/FTCppp discrimination in comparison to Polγ *in vitro*	Koczor and Lewis, 2010; Brown et al., 2011

a *The Polθ carboxyl-terminal polymerase domain has been crystalized inserting ddAppp opposite a template abasic site (Zahn et al., 2015)*.

Nucleoside analogs, including NRTIs, are taken up by cells then phosphorylated to active nucleotide analogs by intracellular kinases (Zhu et al., 2000). Nucleoside kinases such as DCK, CMPK1, and nucleoside diphosphate kinases (NME) act on NRTIs like ddC and perform the first, second, and third phosphorylation steps respectively generating the active NtRTI in the cytoplasm ex. ddCppp, where ppp represent the triphosphate (Liyanage et al., 2017). NtRTIs can then be imported into mitochondria and could compete with native nucleotides at DNA polymerase active sites to inhibit mtDNA replication through chain termination and persistence in the mitochondrial genome. Unlike natural deoxyribonucleotide triphosphate substrates, and with the exception of FIAU, NtRTIs are chain terminators that lack the 3′ hydroxyl group and therefore cannot be extended by a polymerase once incorporated into DNA. Therefore, if these analogs are not removed from DNA, replication will stall (Figure 1B).

## Clinical evidence for NRTI disruption of mtDNA replication

In clinical trials drugs that showed promise in AIDS therapy, such as fluoro-dideoxyadenosine (FDDA), or in the treatment of chronic hepatitis B infection, such as FIAU, toxicity was reported affecting peripheral nerves, liver, skeletal, and cardiac muscle (Lewis et al., 2001). Toxicity to mitochondria was so severe that hepatic failure and death in some patients necessitated discontinuation of their use (McKenzie et al., 1995). One long-term AZT use study of HIV-positive patients concluded that AZT treatment caused toxic mitochondrial myopathy (Dalakas et al., 1990). In a follow-up study investigating mtDNA content in muscle biopsies, mitochondrial genome depletion was discovered in all HIV-positive patients who were treated with AZT and who displayed myopathy and ragged-red fibers in comparison to controls (Arnaudo et al., 1991). Another study investigated HIV-positive patients who developed neuropathy 6–10 weeks after starting ddC and this investigation found mitochondrial alterations and significantly reduced mtDNA copy number in nerve biopsy specimens (Dalakas et al., 2001). These and other observations led to the Polγ dysfunction hypothesis. Hypothetically, poisoning of Polγ would lead to decreased mtDNA, increased mitochondrial stress due to compromised OXPHOS (as OXPHOS subunits are encoded by mtDNA), increased cellular energy depletion (due to diminished ATP pools), and acquired mitochondrial disease phenotypes (Koczor and Lewis, 2010). Key side effects of NRTIs are summarized in Table 2 and (Koczor and Lewis, 2010). Support for the Polγ dysfunction hypothesis comes from cell culture and biochemical work discussed below.

## Evidence for polγ-mediated NRTI toxicity from biochemical studies

Polγ-mediated NRTI mitochondrial toxicity requires that analogs be metabolized to NtRTIs, imported into mitochondria then incorporated into mtDNA and persist there to block further genome replication events. Support for NRTI toxicity caused by inhibition of Polγ DNA polymerase activity comes from extensive biochemical evidence. Pre-steady and steady-state enzyme kinetic analyses have demonstrated that Polγ is able to incorporate various anti-retroviral NtRTIs (Martin et al., 1994; Johnson et al., 2001; Lim and Copeland, 2001; Brown et al., 2011). NtRTIs that have been tested *in vitro* for incorporation into nascent DNA by Polγ include: ddCppp, ddTppp, d4Tppp, ddAppp (the active form of ddI), (+) and (−)3TCppp, PMPApp (PMPA triphosphate), AZTppp, CBVppp (the active form of ABC), and FIAUppp. These biochemical studies agree that Polγ incorporates NtRTIs during DNA replication; however, the efficiency of analog incorporation is variable among the NtRTIs that have been examined. Polγ incorporates ddCppp, ddAppp (ddI), and d4Tppp analogs most efficiently while 3TCppp, PMPApp, AZTppp, and CBVppp (ABC) are modestly incorporated into DNA. Steady-state and pre-steady-state kinetics have also demonstrated that FIAUppp is strongly incorporated by Polγ (Lewis et al., 1996; Johnson et al., 2001). Mitochondrial toxicity, therefore, may be acquired due to a block in mtDNA replication if chain-terminating NtRTIs cannot be removed. Indeed, biochemical evidence has shown that Polγ does not efficiently proofread NtRTIs incorporated into DNA. Pre-steady-state measurements have demonstrated that a ddCp (ddC monophosphate) incorporated into the 3′-end of a DNA oligonucleotide annealed to a DNA template essentially cannot be removed by Polγ proofreading activity even after 12-h incubations with the DNA duplex (Johnson et al., 2001). The remaining NtRTIs analyzed for exonucleolytic removal had slow rates of excision and it has been estimated that the half-life of the reaction to remove (+)3TCp or (−)3TCp is ~1 min (Feng et al., 2001). The rate of NtRTI excision could be detrimental *in vivo* by slowing the mtDNA replication machinery. If Polγ dissociates from mtDNA prior to cleaving an incorporated nucleotide analog then replication would be terminated. When PMPA-terminated DNA substrate was tested for excision in the presence of trap DNA, no Polγ exonuclease activity was detected (Johnson et al., 2001). This finding suggests that NtRTI-containing duplex DNA is released from Polγ prior to NtRTI excision and perhaps a similar mechanism could happen *in vivo* with many copies of mtDNA. Similar findings of slow rates of NtRTI excision were observed utilizing steady-state analyses. Additionally, Polγ exonuclease activity was inhibited at *in vivo* concentrations of the AZTppp phosphorylated intermediate AZT monophosphate, AZTp (Lim and Copeland, 2001). Perhaps *in vivo* intracellular levels of AZTp allow for binding of the analog to the exonuclease active site and lower Polγ's fidelity by blocking proofreading.

In 2015 crystal structures of Polγ-DNA replication complexes separately bound to ddCppp or to the natural substrate dCTP were solved (Szymanski et al., 2015). Within the DNA polymerase active site the side chain of the p140 Y951 residue stacks with the incoming ddCppp nearly identically to the natural dCTP substrate. The ribose sugar moieties of both nucleotides are located 3.5 Å from the p140 Y951 hydroxyl group. In support of the p140 Y951 residue being the likely cause of ddCppp toxicity, a biochemical study demonstrated that mutation of Y951 to phenylalanine maintains DNA polymerase activity but renders p140 Y951F almost completely incapable of incorporating ddCppp, CBVppp, 3TCppp, and d4Tppp (Lim et al., 2003). The p140 Y951F had a 2,400-fold increase in dCTP/ddCppp discrimination relative to wild-type p140. Therefore, the substitution of the smaller phenylalanine side chain in the p140 Y951F variant must influence the structure such that ddCppp is excluded from the DNA polymerase active site and not readily incorporated into DNA.

Variability in mtDNA depletion has been observed in HIV-positive patients treated with NRTIs and may result from a difference in treatment times or from genetic variations that have increased susceptibility to NRTIs or both. A homozygous mutation encoding p140 R964C was identified in a 34-year-old HIV-infected woman with a history of lactic acidosis induced by d4T treatment (Yamanaka et al., 2007). Recombinant p140 R964C displays 14% polymerase activity relative to WT p140. Additionally, a patient-derived p140 R964C lymphoblastoid cell line (LCL) cultured with d4T displays mtDNA depletion relative to a WT LCL suggesting p140 R964C is associated with severe lactic acidosis induced by NRTI use. A pre-steady state analysis of Polγ holoenzyme harboring the p140 R964C variant determined that the substitution caused a 33% reduction in dTTP incorporation efficiency and a 3-fold decrease in dTTP/d4Tppp discrimination relative to WT suggesting p140 R964C has a higher propensity to incorporate d4Tppp (Bailey et al., 2009). The p140 R964 residue is located in close proximity to the DNA polymerase active site. One explanation for the mechanism of increased d4Tppp incorporation is that the p140 R964C substitution modulates active site access increasing binding to d4Tppp. Also, a heterozygous mutation (C>T 2857/p140 R953C) was identified in an HIV-infected patient undergoing antiretroviral therapy who displayed mitochondrial toxicity and mtDNA depletion (Li et al., 2016). The recombinant R953C Polγ holoenzyme displayed an 8-fold weakened ability to bind to dCTP and a 4-fold decrease in its ability to discriminate between dCTP and (−)-3TCppp relative to WT. Molecular modeling revealed that a cysteine substitution at position 953 in p140 could abolish interactions between p140 side chain residues in the active site thereby reducing the binding of an incoming nucleotide. In another case-control study examining the relationship between p140 E1143D/G substitutions, lipodystrophy, and d4T treatment it was concluded that HIV-infected patients harboring an E1143D/G variant are 4-fold more likely to develop lipodystrophy and if treated with d4T the risk of developing lipodystrophy increased (Chiappini et al., 2009).

## Evidence for NRTI disruption of mtDNA replication from cell culture and animal studies

Support for intracellular NRTI mitochondria toxicity mediated by disruption of mtDNA replication comes from observations that primary and immortalized cell lines undergo mtDNA depletion upon exposure to various NRTIs. Table 3 lists examples of human cell lines exposed to various nucleoside analogs in tissue culture. In some reports, mtDNA depletion was so severe cell lines became rho zero completely lacking mtDNA. These findings are similar to what has been reported with LA9 mouse cells exposed to ddC (Brown and Clayton, 2002) and with treating human cell lines with the mtDNA replication inhibitor ethidium bromide, EtBr (King and Attardi, 1996). Low concentrations of EtBr either partially or completely inhibit maintenance of the negatively supercoiled circular mitochondrial genome but not nuclear DNA (nDNA). EtBr binds better to negatively supercoiled substrates than to positively supercoiled ones and might enhances topoisomerase-mediated cleavage of negatively supercoiled DNA; therefore, EtBr may act as a topoisomerase topological poison (Gentry et al., 2011). In agreement with Polγ biochemical analyses, treatment of human cell lines with several nucleoside analogs typically duplicate the finding that ddC causes the most severe inhibition of mtDNA replication as indicated by mtDNA depletion. In an animal study investigating AZT exposure by administering the drug in drinking water to rats, transmission electron microscopy revealed widespread mitochondrial alterations in the heart following 35 days of treatment with 1 mg/ml AZT (Lewis et al., 1991). In another 4-month study investigating the treatment of BALB/C mice with ddI, d4T, AZT, or 3TC, and with the exception of liver tissue from mice treated with 3TC, mtDNA depletion was reported in liver, muscle, and cortical neurons. Also, cortical neurons isolated from mice treated with ddI, d4T, and 3TC were reported to harbor an increased level of mtDNA deletions (Zhang Y. et al., 2014).

**Table 3:** NRTIs that disrupt mtDNA maintenance in human cell lines.

**Cell line**	**Source of cell line**	**Nucleoside analog or agent studied^a^**	**Effect on mtDNA maintenance**	**Treatment time**	**References**
Molt-4	T lymphoblast	AZT, d4T, FLT, 935U83, FIAU, 524W91, 3TC, ddC, ddI	ddC and FLT, mtDNA depletion and cell death; d4T caused mtDNA depletion; FIAU did not alter ratio of mtDNA to nDNA but was cytotoxic; 524W91, AZT, 935U83 no detectable affect on mtDNA or cell growth	5 days (FIAU and ddC), 6 days (d4T), rest 7 days	Martin et al., 1994
HepG2	Hepatocellular carcinoma	PMPA, 3TC, ABC, ddC, ddI, d4T, and AZT	PMPA, 3TC, and ABC had no detectable effects on mtDNA levels; ddC > ddI > d4T > AZT depletion of mtDNA	9 days	Birkus et al., 2002
Primary SkMC	Skeletal muscle cells	PMPA, 3TC, ABC, ddC, ddI, d4T, and AZT	PMPA, 3TC, ABC, AZT had no detectable effects on mtDNA levels; ddC > ddI > d4T depletion of mtDNA	9, 18, and 21 days	Birkus et al., 2002
Primary RPTECs	Renal proximal tubule epithelial cells	PMPA, ddC, ddI, d4T, and AZT	PMPA & AZT had no detectable effects on mtDNA levels; ddC > ddI > d4T depletion of mtDNA	12 and 21 days	Birkus et al., 2002
Lymphocytes	Primary peripheral blood lymphocytes	ddC, ddI, d4T, AZT	ddI > ddC > d4T deplete mtDNA; AZT did not affect mtDNA but increased lactic acid production and reduced cell counts	10 days	Setzer et al., 2005
Lymphoblastoid cell line	Blood lymphocytes transformed with the Epstein Barr Virus	ddC	MtDNA depletion down to 20% of untreated cells	15 days	Rocher et al., 2008
HCA2-htert	Fibroblast cell line immortalized by over-expression of human telomerase	ddC	Extreme mtDNA depletion	8 days	Ashley et al., 2005
KP hMSC	Immortalized mesenchymal/stromal cell line	EtBr, AZT, d4T	MtDNA depletion EtBr > d4T > AZT	10 days	Fernández-Moreno et al., 2016
3a6 hMSC	Immortalized mesenchymal/stromal cell line	EtBr, AZT, d4T	MtDNA depletion d4T > EtBr; AZT no detectable mtDNA depletion	6 (d4T), 9 (AZT), or 10 (EtBr) days	Fernández-Moreno et al., 2016
HeLa	Cervical cancer cells	ddC	MtDNA depletion	3 days	Jemt et al., 2015
CEM	Leukemia cell line	ddC, d4T, ddI	MtDNA depletion, potencies in reducing cell viability, mtDNA content and normal mitochondrial morphology were ddC > d4T > ddI	4 days	Medina et al., 1994

a *FLT, 3′-fluoro-3′-deoxythymidine; 935U83, 3′-fluoro-2′, 3′-dideoxy-5-chlorothymidine; 524W91, [(−) FTC], (−)-β-L-2′, 3′-dideoxy-5-fluoro-3′-thiacytidine; EtBr, ethidium bromide*.

## Other potential mechanisms of NRTI toxicity

Other mechanisms of NRTI toxicity include increased frequency of mtDNA mutations (perhaps from an altered Polγ function), enhanced oxidative stress, and competition with endogenous nucleotides for kinases required to phosphorylate and activate them thereby lowering the *in vivo* concentrations of nucleotides available to replicate mtDNA (McKee et al., 2004). The recent discovery of other cellular DNA polymerases localizing to human mitochondria also has implications for NRTI toxicity as these enzymes may incorporate analogs. Purified Polβ is considerably sensitive to NtRTIs including d4Tppp and ddCppp (Martin et al., 1994; Pelletier et al., 1994) and compared to Polγ is less selective for and can incorporate AZTppp, PMPApp, L-FTCppp, and L-3TCppp (Table 2 and Brown et al., 2011). In the nucleus Pols alpha (α), delta (δ), and epsilon (ε) harbor strong nucleotide selection mechanisms and are less likely to incorporate NtRTIs (Brown et al., 2012). Incorporation of NtRTIs by Polβ within the organelle would be complicated by (1) the sensitivity of analog incorporation by Polγ and (2) the lack of Polβ proofreading activity, which would likely contribute to NtRTI persistence within mtDNA. Figures 1A,B highlight key steps in BER and mtDNA replication that could be negatively affected by NtRTIs. Finally, the mitochondrial localization of DNA repair polymerases with flexible active sites could allow for accommodation of nucleotide analogs and contribute to unwanted insertion of chain terminators. Pre-steady-state analyses of PrimPol NtRTI incorporation kinetics revealed effective incorporation of CBVppp, followed by ddCppp > ddAppp > AZTppp while d4Tppp, 3TCppp, PMPApp, and FTCppp were not readily incorporated. From this study, it was determined that CBVppp is actually a better substrate for PrimPol than for HIV-RT which may help to explain life-threatening sensitivity to this analog in some patients (Mislak and Anderson, 2015).

## Evidence for POLRMT-mediated AVRN toxicity from biochemical and cell culture studies

POLRMT directs polycistronic transcription from three promoters the heavy-strand promoter 1 (HSP1), the HSP2, and the light-strand promoter, LSP (Lodeiro et al., 2012; Figure 1C). The two mtDNA strands are named heavy (H) and light (L) based on the ability to separate them on alkaline cesium chloride buoyant density gradients (Kasamatsu and Vinograd, 1974). RNA polymerase enzymes known as primases synthesize RNA primers required for initiation of DNA replication. Evidence supporting the role of human POLRMT as the mtDNA primase comes from the identification of primers located adjacent to nascent H-strands isolated from human KB cell mitochondria (Chang and Clayton, 1985), from *in vitro* experiments demonstrating that POLRMT has primase activity (Wanrooij et al., 2008), and from the observation that replicating mtDNA obtained from mouse embryonic fibroblasts, and lacking RNaseH1, retain unprocessed primers at origins of replication (Holmes et al., 2015). The 5′-end of RNA primers that have been mapped to the LSP therefore likely serve to initiate synthesis of nascent H-strand mtDNA (Chang and Clayton, 1985; Figure 1B). Consequently, mtDNA replication is likely dependent on mitochondrial transcription.

Sofosbuvir is an antiviral uridine analog inhibitor of hepatitis C virus (HCV) RNA-dependent RNA polymerase (HCV non-structural protein 5B, NS5B) currently approved for use to treat patients with HCV infections. A number of reports have described the potential use of other antiviral ribonucleosides (AVRNs) as anti-viral and anti-cancer agents; however, many of these AVRNs have had adverse toxic effects when administered to patients and did not pass clinical trials or gain FDA approval (Arnold et al., 2012a,b). For example, the AVRN analog BMS-986094 developed to treat HCV infection did not pass phase II development after nine patients became hospitalized and one died (Mislak and Anderson, 2015). Utilizing a POLRMT *in vitro* biochemical system to measure substrate utilization a panel of more than 10 AVRN analogs were investigated that contained moieties found in past and lead anti-HCV non-obligate chain terminators (Arnold et al., 2012a). Non-obligate chain terminators are AVRNs containing a 3′-OH yet prevent viral RNA elongation. Except for one analog, all AVRN triphosphates (AVRNts) investigated were readily utilized by POLRMT as off-target substrates and five analogs were strong non-obligate chain terminators of POLRMT RNA elongation. Utilizing the human hepatoma cell line, Huh-7, the panel of AVRNs were metabolized to active triphosphates, presumably by intracellular kinases, and the levels of the triphosphate forms varied from less than 0.15 μM to 3.5 mM. Cellular evidence for AVRNs being used as substrates by POLRMT was demonstrated using Huh-7 cells pre-treated for 24 h with EtBr to suppress mitochondrial transcription then cells were exposed to AVRNs for 1, 2, and 3 days. Mitochondrial transcription was impaired in cells exposed to 2′-C-methyladenosine, 6-methylpurine-riboside, and 4′-azidocytidine. This study demonstrated that toxic effects of AVRNs might result from inhibition of the mitochondrial transcription machinery and mtDNA gene expression (Arnold et al., 2012a). Due to the close coupling of mitochondrial transcription and mtDNA replication, prolonged exposure to AVRNs might also affect mtDNA maintenance, Figures 1B,C.

## Other reports of drugs with off-target effects on mtDNA maintenance

Four human cellular topoisomerases localize to mitochondria: TOP1mt, TOP2α, TOP2β, and a TOP3α long isoform (Zhang H. et al., 2014; Pommier et al., 2016). Tamoxifen a drug used to prevent breast cancer, tacrine a drug used to treat Alzheimer's disease, and a fluoroquinolone broad-spectrum antibiotic, have all been hypothesized to have off-target effects on mitochondrial topoisomerases (Lawrence et al., 1996; Mansouri et al., 2003; Larosche et al., 2007; Nadanaciva et al., 2010; Begriche et al., 2011). Mice separately treated for 28 days with tamoxifen and tacrine displayed mtDNA depletion and both of these drugs were demonstrated to inhibit *in vitro* topoisomerase-mediated plasmid DNA relaxation (Mansouri et al., 2003; Larosche et al., 2007). The fluoroquinolone ciprofloxacin, an inhibitor of bacterial type II topoisomerase DNA gyrase, was reported to induce double-strand mtDNA breaks when mouse L1210 cells were exposed to various concentrations of the drug (Lawrence et al., 1996). The pyrrole alkaloid lamellarin D and the chemotherapy drug doxorubicin have both been shown to poison mitochondrial and nuclear topoisomerases (Pommier et al., 2016).

Menadione (vitamin K3, VK3) has been demonstrated to inhibit the growth of human cancer cell lines derived from various tissues and induces an increase in ROS leading to apoptosis. In an *in vitro* biochemical assay VK3 selectively inhibited Polγ DNA polymerase and RT activities but did not inhibit the activity of other DNA polymerases tested including Pols α, β, δ, ε, eta (η), iota (ι), kappa (κ), and lambda (λ). The authors proposed that suppression of mtDNA replication and repair could trigger ROS production leading to apoptotic cell death (Sasaki et al., 2008). Although the neurotoxicant 1-methyl-4-phenylpyridinium ion (MPP+) does not directly inhibit the catalytic activity of Polγ, MPP+ was reported to cause mtDNA depletion by destabilizing the mtDNA displacement-loop, a mtDNA replication intermediate, thereby inhibiting mitochondrial genome replication (Umeda et al., 2000). Acetaminophen (APAP or paracetamol) is a commonly used over the counter drug used for fever and pain relief. Mice treated with 300 mg/kg of acetaminophen had mtDNA depletion as quantitated using a slot blot hybridization technique (Cover et al., 2005). The depletion is likely due to mtDNA stand breaks caused by the production of ROS, reactive nitrogen species (RNS), and other reactive metabolites followed by rapid degradation of damaged mtDNA by endogenous mitochondrial endonucleases (Begriche et al., 2011). Troglitazone is an anti-inflammatory and anti-diabetic drug that was withdrawn from the market due to serious hepatotoxicity. Primary human hepatocytes exposed to troglitazone had increased mtDNA depletion, decreased ATP production, and decreased cellular viability (Rachek et al., 2009). ROS and oxidative stress were hypothesized to be the source of mtDNA depletion causing mtDNA strand breaks and cytotoxicity and treatment with *N*-acetyl cysteine (NAC), a known ROS scavenger, reduced the troglitazone-induced cytotoxicity. Cisplatin is a platinum-based FDA-approved chemotherapeutic known to damage nDNA by forming inter-strand crosslinks. Patients treated with platinum-based compounds often display peripheral neuropathy, which may result from damage to dorsal root ganglion neuronal mtDNA (Cline, 2012). *In vitro* work has demonstrated cisplatin or oxaliplatin block Polγ DNA synthesis. Furthermore, cisplatin has been demonstrated to inhibit rat neuronal mtDNA replication and mitochondrial transcription (Vaisman et al., 1999; Podratz et al., 2011; Cline, 2012).

## Targeting mtDNA maintenance to kill cancer cells

Cancer cells display uninhibited DNA replication; therefore, DNA polymerases and DNA repair proteins have been exploited as therapeutic targets to combat certain types of cancer (Lange et al., 2011; Somasagara et al., 2017). NRTI-sensitive mitochondrial DNA polymerases afford a unique opportunity to target cancer cell mitochondria as certain cancers have an increased reliance on OXPHOS and nDNA polymerases are less sensitive to NRTI inhibition (Martin et al., 1994; Liyanage et al., 2017). In a study comparing normal hematopoietic cells to a panel of 542 primary acute myeloid leukemia (AML) samples, it was recently discovered that 55% of the AML samples had increased mtDNA biosynthesis gene expression. Upregulated genes included *POLG, POLG2, POLRMT, Twinkle, TFAM, SSBP1, DGUOK, TK2*, nucleotide transporters (*SLC25A33, SLC25A36*, and *SLC29A3*) and nucleoside kinases (*CMPK1* and *NME1-NME2*). When treated with ddC AML cells preferentially activated the analog and blocked mtDNA replication and OXPHOS in comparison to hematopoietic cells. Cytotoxicity was preferentially activated in NRTI-treated AML cells and an AML animal model treated with low doses of ddC (35 and 75 mg/kg/day over 11 days) resulted in decreased mtDNA, decreased mtDNA-encoded cytochrome oxidase subunit 2 (COX II), and induced tumor regression without apparent toxicity (Liyanage et al., 2017).

Targeting mtDNA maintenance has also been exploited to treat cancer cell lines with a mitochondrial-targeted cisplatin (Marrache et al., 2014). Nucleotide excision repair (NER) machinery repairs cisplatin-nDNA adducts; however, mitochondria lack NER machinery to deal with this type of damage. Most cancer cells have an increased mitochondrial membrane potential relative to non-cancer cells and triphenylphosphonium (TPP) cations are targeted to mitochondria due to their size, lipophilic properties, and delocalized positive charge. An engineered TPP-tagged cisplatin, Platin-M, caused increased cytotoxicity relative to cisplatin only treatment in several cancer cell models: cisplatin-resistant A2780/CP70 ovarian cancer, prostate cancer PC3 (inherently resistant to cisplatin therapy), and SH-SY5Y neuroblastoma cells. Furthermore, encapsulating Platin-M into specialized nanoparticles enhanced cytotoxicity. SH-SY5Y cells treated with Platin-M and Platin-M encapsulated in nanoparticles were annexin V-positive and propidium iodide-negative, indicative of early apoptosis. Treatment with both Platin-M and Platin-M encapsulated in nanoparticles weakened mitochondrial citrate synthase activity and diminished bioenergetic parameters: spare respiratory capacity, coupling efficiency, and basal respiration. PC3 cells treated separately with cisplatin, Platin-M, and Platin-M encapsulated inside of nanoparticles were subjected to subcellular fractionation then platinum concentrations in various fractions were quantified. Cells treated with Platin-M and Platin-M encapsulated in nanoparticles, contained platinum-mtDNA adducts while cells treated with cisplatin contained mostly platinum-nDNA adducts. These findings support that cisplatin is likely released from Platin-M within mitochondria then binds to mtDNA and inhibits replication.

## Conclusions

Evaluation of antibiotic and antiviral mitochondrial exposures using biochemistry and human cell line and animal models is an important consideration for determining drug toxicity because the complex mitochondrial network harbors multiple copies of OXPHOS complexes and mtDNA that may cause a slow response to these agents. Chronic exposures to drugs may result in long-term mtDNA and OXPHOS depletion. NRTIs may have tissue-specific toxicities such as skeletal- and cardio-myopathies, peripheral neuropathy, and others (Table 2 and Lewis et al., 2001). Side effects may limit NRTI use in some individuals, cause organ failure and death in others or may only result in minor discomfort (Koczor and Lewis, 2010). Gene variations (like those seen in *POLG* encoding p140 R964C, R953C, and E1143D/G) may exacerbate mitochondrial disease-like phenotypes in HIV-infected patients treated with NRTIs. Also, valproic acid has been demonstrated to induce liver failure in autosomal recessive *POLG* disease but may not be as toxic in autosomal dominant disease. However, due to the potential for valproic acid to cause death by liver toxicity experts recommend avoiding this drug (Saneto and Naviaux, 2010). *POLG* is a highly polymorphic gene and the association between disease-causing and non-disease causing substitutions are often unclear and dependent on other complex factors. How other drugs or environmental factors interact with various genetic variant backgrounds (so-called ecogenetic single nucleotide variants, ESNVs) and contribute to mitochondrial disease manifestation is poorly understood (Saneto and Naviaux, 2010; Zolkipli-Cunningham and Falk, 2017). Do ESNVs predispose individuals to mitochondrial dysfunction via pharmacological or environmental toxicants while individuals harboring other polymorphism remain resistant? ESNV-environment interaction is an important area for future mitochondrial disease and mtDNA maintenance research. Evidence suggests mitochondria are targets of environmental toxicants that disrupt mtDNA maintenance and chemical exposures may cause increased and decreased mtDNA copy number. At low doses, oxidative stress stimulates mtDNA replication but at high doses mtDNA depletion. Polycyclic aromatic hydrocarbons cause more damage to mtDNA than to nDNA and a compilation of studies comparing nDNA to mtDNA damage following chemical exposure has been reviewed (Meyer et al., 2013).

Evidence suggests five DNA polymerases localize to human cell mitochondria: Polγ, Polβ, PrimPol, Polθ, and Pol ζ. *In vitro* biochemistry measuring substrate binding and incorporation lends strong support to Polγ, Polβ, and PrimPol being off-targets for nucleotide analogs (Pelletier et al., 1994; Szymanski et al., 2015). Additionally, the Polθ carboxyl-terminal polymerase domain has been crystallized in a translesion DNA synthesis mode inserting ddAppp opposite a template abasic site (Zahn et al., 2015). Comparative investigations of mtDNA polymerase enzyme kinetics and determination of crystal structures with and without lesions will assist in our understanding of the spectrum of mtDNA polymerase toxicity. The overarching goal is that these structure-function studies will assist with designing novel antiviral analogs with higher specificity to viral polymerases and less mitochondrial off-target effects. In mice, mitochondrial Polβ was undetectable in heart, liver, and muscle but was present in organelles obtained from brain and kidney (Sykora et al., 2017). Potential questions for future research include: (1) How are the other newly identified mtDNA polymerases distributed among human organs and tissues and do they associate with other components of the mtDNA repair or replication machinery? Knowledge of the distribution of mtDNA polymerases within human tissues may assist with the prediction of tissue-specific toxicant effects. (2) Could knowledge of mtDNA polymerases within different tissues be exploited to treat certain types of cancers with NRTIs? (3) What analogs and toxicants are incorporated by the newly identified mtDNA polymerases? And (4) Do ESNVs exist in any other genes of interest required for mtDNA maintenance? Current next-generation sequencing technologies and continued research utilizing *in vitro* biochemistry and model systems such as human cell lines and mice will be essential to answer these questions and will be necessary for future investigations of mitochondrial dysfunction and disease.

## Author contributions

The author confirms being the sole contributor of this work and approved it for publication.

### Conflict of interest statement

The author declares that the research was conducted in the absence of any commercial or financial relationships that could be construed as a potential conflict of interest.
